# Intestinal tuberculosis revealed by acute intestinal obstruction: a case report and review of the literature

**DOI:** 10.1093/jscr/rjaf546

**Published:** 2025-07-18

**Authors:** Moctar N Fodiya, Nassiba O Touhami, Omar Mkira, Habib O Dato, Sabrine Derqaoui, Ahmed Jahid, Rachid Sani, Omar Belkouchi

**Affiliations:** Department of General Surgery, Ibn Sina University Hospital Center, Mohamed V University, Avenue Hafiane Cherkaoui, X4MV+GJ, Rabat, Morocco; Department of General Surgery, Faculty of Health Sciences of Abdou Moumouni University, G33M+H5V, Niamey, Niger; Department of Pathology, Ibn Sina University Hospital Center, Mohamed V University, Avenue Hafiane Cherkaoui, X4MV+GJ, Rabat, Morocco; Department of General Surgery, Ibn Sina University Hospital Center, Mohamed V University, Avenue Hafiane Cherkaoui, X4MV+GJ, Rabat, Morocco; Department of General Surgery, Ibn Sina University Hospital Center, Mohamed V University, Avenue Hafiane Cherkaoui, X4MV+GJ, Rabat, Morocco; Department of Pathology, Ibn Sina University Hospital Center, Mohamed V University, Avenue Hafiane Cherkaoui, X4MV+GJ, Rabat, Morocco; Department of Pathology, Ibn Sina University Hospital Center, Mohamed V University, Avenue Hafiane Cherkaoui, X4MV+GJ, Rabat, Morocco; Department of General Surgery, Faculty of Health Sciences of Abdou Moumouni University, G33M+H5V, Niamey, Niger; Department of General Surgery, Ibn Sina University Hospital Center, Mohamed V University, Avenue Hafiane Cherkaoui, X4MV+GJ, Rabat, Morocco

**Keywords:** surgical emergency, acute intestinal obstruction, extra-pulmonary tuberculosis, intestinal tuberculosis

## Abstract

Tuberculosis is a major public health problem. It is an infectious disease present in every country in the world and can occur at any age, with a higher incidence in developing countries. Among the extra-pulmonary forms, the intestinal form is rare and often misdiagnosed and/or lately at the stage of complications such as occlusion, stricture, perforation, or hemorrhage. Unfortunately, this contributes to the increased mortality and morbidity associated with this benign disease. We report the case of a 78-year-old patient who presented to our department with an acute intestinal obstruction, secondary to an ileal mass. He had undergone bowel resection of the mass and hand sewn end-to-end anastomosis. Histopathological examination of the specimen confirmed intestinal tuberculosis.

## Introduction

Tuberculosis (TB) is an infectious disease caused by *Mycobacterium tuberculosis*. It is one of the first cause of death among infectious diseases worldwide, ahead of human immunodeficiency virus/acquired immunodeficiency syndrome (HIV AIDS), and the ninth most common cause of death [1]. Although the lungs are the common site of this disease, it can affect several organs (genitourinary tuberculosis, meningeal tuberculosis, peritoneal tuberculosis, pericardial tuberculosis, tuberculous lymphadenitis, cutaneous tuberculosis, tuberculosis of bones and joints, gastrointestinal tuberculosis, tuberculosis of the liver, etc.) [2]. The gastrointestinal localization, although rare, is not exceptional, especially in endemic areas such as Morocco, but it is often lately diagnosed.

## Case report

The patient was a 78-year-old male, with no medical history. He has no familial cases of recent tuberculosis infection. The patient presented to our department, with an acute bowel obstruction.

Physical examination found a very distended abdomen.

**Figure 1:**
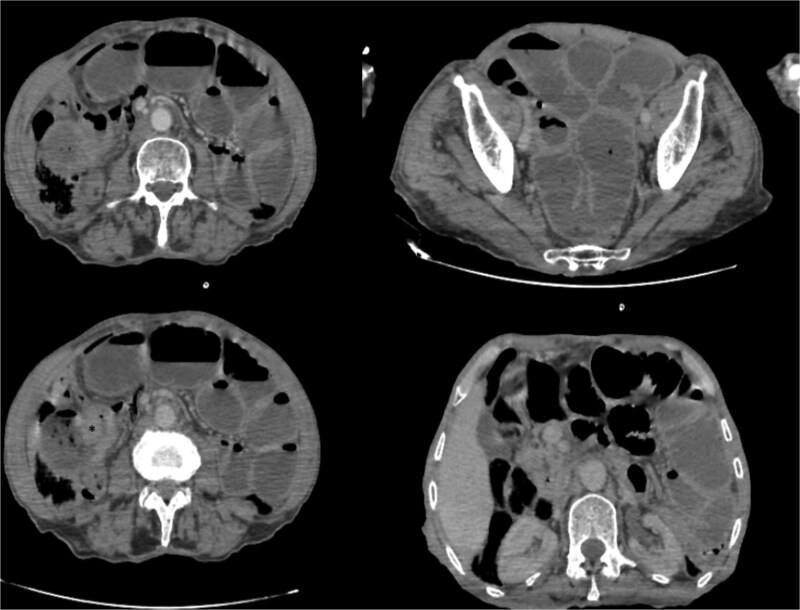
Abdominal computed tomography scan showing dilated jejunal loops; (*): Ileal mass.

Biological tests were normal, but C-reative protein levels were slightly elevated at 24.20 mg/l (reference value <5 mg/l). Abdomino-pelvic scan, showed a dilated jejunum and ileum up stream of an ileal wall thickening, with minimal free fluid in the pelvis (Fig. 1).

**Figure 2:**
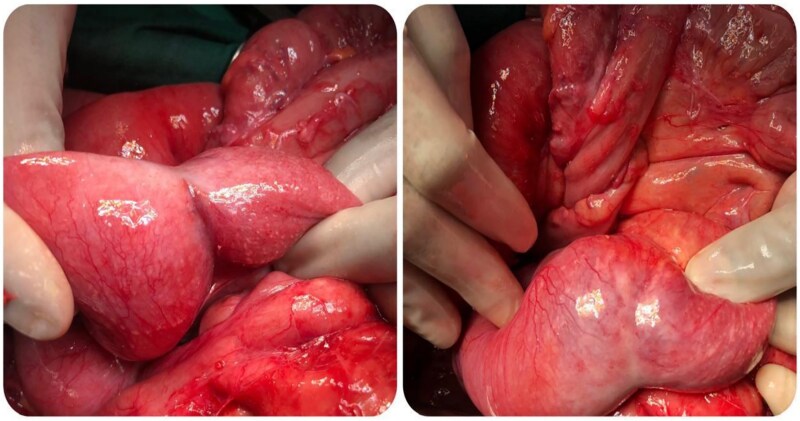
Intraoperative images of the mass.

Laparotomy by a midline incision was performed. Exploration revealed a distended small bowel over an ileal mass located at 80 cm from the ileocaecal junction, some pelvic fluid and some adhesions between bowel loops (Fig. 2). There was no liver metastasis or peritoneal carcinomatosis. Excision of the mass was performed, followed by a hand sewn end to end ileo-ileal anastomosis.

The post-operative course was uneventful, and the patient was discharged on postoperative day 5. The cyto bacteriological examination of the peritoneal fluid was normal.

**Figure 3:**
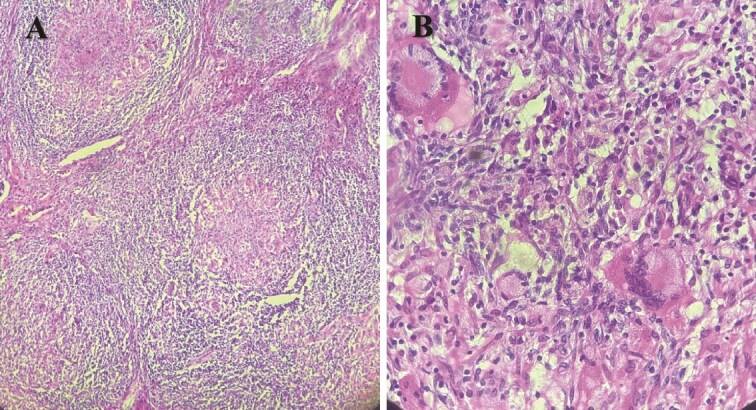
Histological images showing granulomatous inflammatory changes with epithelioid giant cells, as well as early caseous necrosis.

Macroscopique examination of the speciman schowed revealed inflammatory granulomatous epithelio-giganto-cellular changes with caseous necrosis, suggestive of tuberculosis (Fig. 3).

After the result of the anatomo-pathological examination of the surgical specimen, the patient was referred to the pneumo-phthisiology department where a first-line anti-tuberculosis treatment (2RHZE/4RH protocol) was applied, in accordance with the directives of the “Programme national de lutte contre la tuberculose” (PNLAT).

## Discussion

According to the World Health Organization, 20% of tuberculosis cases worldwide are extra-pulmonary. In Morocco, the number of new cases of the extrapulmonary form has increased by 2.3% per year over the last four decades [3].

The intestinal form can affect all parts of the digestive tract. In our case, the mass was located 80 cm from the ileocaecal junction, although the most common location in the digestive tract according to the literature is the ileo-caecal region (an area rich in lymphoid tissue) [4].

Several pathophysiological mechanisms may explain the occurrence of the intestinal form [5]:

- Haematogenous dissemination of BAAR from an active pulmonary site;
- Ingestion of infected saliva by patients with active pulmonary tuberculosis;
- Ingestion of contaminated milk;
- contamination by contiguity from an adjacent affected organ.

This form affects all sexes [6], but some male [7] or female [8] predominance has been reported in the literature. Our patient was 78 years old, whereas this form of the disease mainly affects young people aged between 20 and 40 [9].

The clinical manifestations in this form are not specific; they are the classic signs common to all intestinal pathologies (such as inflammatory bowel disease, cancer or other infectious intestinal diseases), which makes diagnosis difficult. However, N. Bel Kahla *et al.* found that the main symptom was abdominal pain, followed by asthenia (90% and 54%, respectively) which our patient presented [10].

At the time of diagnosis, the disease is often presented at a complicated stage such as bowel obstruction (20% of cases) or hemorrhage or perforation [11, 12].

Although Koch’s bacilli (BK) were not identified in our patient’s peritoneal fluid, their detection can be used to establish the diagnosis. Blood tests may be normal or show inflammatory anemia and lymphocytosis. The sedimentation rate is often accelerated, and the tuberculin skin test (TST) may be positive [13].

As clinical and radiological signs are not specific to the disease but it contributes to the diagnosis. Chest X-rays can be used to look for associated pleuropulmonary localizations. Abdominal ultrasound and abdominal computed tomography may show an intestinal thickening, lymph nodes, and other solid organ lesions, it can also be used for cyto puncture or guided biopsies. Entero-scanner and entero-MRI are more effective in detecting small bowel involvement in the form of parietal thickening with or without luminal narrowing [14].

However, it is the histopathological examination that confirms the diagnosis of tuberculosis by revealing epithelioid and gigantocellular granulomas associated with caseous necrosis. These granulomas are present in our patient but their frequency varies according to other studies; N. Bel Kahla *et al.* found ⁓27% of cases in their case series [10].

Treatment of intestinal tuberculosis in a complicated setting is surgery, such as bowel obstruction, hemorrhage or perforation. It may also be considered in cases of symptomatic stenosis persisting after antituberculosis drugs. However, surgical treatment would not be as effective as without adjuvant anti-tuberculosis treatment according to the first-line protocol (2RHZ / 4RH for 6 months); or second-line (2SRHZE / 1RHZE / 5RHE for 8 months) in the case of relapse or discontinuing treatment [11].

The prognosis will depend on how fast the patient is treated. The high incidence of postoperative mortality in complicated forms is due to the delay in diagnosis [11].

## Conclusion

Although there has been a rise of extra-pulmonary forms in recent decades, the intestinal form of tuberculosis is rare, and acute intestinal obstruction is even rarer. It should be suspected in the presence of any asthenia associated with suggestive small bowel wall thickening and lymph nodes. Surgical treatment must be combined with anti-tuberculosis drugs for complicated forms. But the pandemic prevention is mandatory, by Bacille Calmette-Guérin (BCG) vaccination, prevention of livestock, and public awareness campaigns.

## References

[1] World Gastroenterology Organisation Global Guidelines. Tuberculose digestive. World Gastroenterology Organisation, March 2021. https://www.worldgastroenterology.org/UserFiles/file/guidelines/digestive_tract_tuberculosis_French_2021.pdf.

[2] Nardell EA , Harvard Medical School. Reviewed/Revised Jul 2022 | Modified Apr 2025. Extrapulmonary Tuberculosis (TB). https://www.msdmanuals.com/professional/infectious-diseases/mycobacteria/extrapulmonary-tuberculosis-tb.

[3] Kingdom of Morocco, Ministry of Health and Social Protection, Programme National de Lutte Antituberculeuse, Service des Maladies Respiratoires Division des Maladies Transmissibles, Direction de l'Epidémiologie et de la Lutte contre les Maladies, Lignes directrices nationales pour le diagnostic et la prise en charge de la tuberculose extra pulmonaire. August 2023. Available on: https://www.sante.gov.ma/Documents/2024/04/Version%20Finale%20Lignes%20Directrices%20TEP%20PNLAT%20Version%20finale%20%20Ao%C3%BBt%202023.pdf.

[4] Epstein D, Mistry K, Whitelaw A, et al. The effect of physiological concentrations of bile acids on in vitro growth of mycobacterium tuberculosis. S Afr Med J 2012;102:522–4. 10.7196/samj.5763 22668954

[5] Horvath KD, Whelan RL. Intestinal tuberculosis: return of an old disease. Am J Gastroenterol 1998;93:692–6. 10.1111/j.1572-0241.1998.207_a.x 9625110

[6] Sharma MP, Bhatia V. Abdominal tuberculosis. Indian J Med Res 2004;120:305–15. 15520484

[7] Leung VK, Law ST, Lam CW, et al. Intestinal tuberculosis in a regional hospital in Hong Kong: a 10-year experience. Hong Kong Med J 2006;12:264–71. 16912352

[8] Muneef MA, Memish Z, Mahmoud SA, et al. Tuberculosis in the belly: a review of forty-six cases involving the gastrointestinal tract and peritoneum. Scand J Gastroenterol 2001;36:528–32. 10.1080/003655201750153412 11346208

[9] Collado C, Stirnemann J, Ganne N, et al. Gastrointestinal tuberculosis: 17 cases collected in 4 hospitals in the northeastern suburb of Paris. Gastroenterol Clin Biol 2005;29:419–24. 10.1016/s0399-8320(05)80796-1 15864206

[10] Kahla NB, Naija N, Ouerghi H, et al. Intestinal tuberculosis: report of 11 cases. J Afr Hépatol Gastroentérol 2010;4:236–40. 10.1007/s12157-010-0203-9

[11] Ben Chaabane N, Ben Mansour W, Hellara O, et al. La tuberculose gastro-intestinale. Hepato Gastro 2012;19:28–35. 10.1684/hpg.2012.0674

[12] Ha HK, Ko GY, Yu ES, et al. Intestinal tuberculosis with abdominal complications: radiologic and pathologic features. Abdom Imaging 1999;24:32–8. 10.1007/s002619900436 9933670

[13] Ghabisha S, Ahmed F, Almohtadi AM, et al. Demographic, clinical, radiological, and surgical outcome of patients with intestinal tuberculosis: a single-center retrospective study. Res Rep Trop Med 2024;15:79–90. 10.2147/RRTM.S465571 39253062 PMC11382657

[14] Aissaoui M, Osmane R, Benrabia A, et al. Tuberculose intestinale. Journal Algérien de Gastroentérologien 2023;**35**:14–26. https://accueil.sahgeed.com/wp-content/uploads/2024/04/JAG35.pdf.

